# Berbamine suppresses intestinal SARS-CoV-2 infection via a BNIP3-dependent autophagy blockade

**DOI:** 10.1080/22221751.2023.2195020

**Published:** 2023-04-17

**Authors:** Alexandra P. M. Cloherty, Anusca G. Rader, Kharishma S. Patel, Jimena Pérez-Vargas, Connor A. H. Thompson, Siobhan Ennis, Masahiro Niikura, Manon E. Wildenberg, Vanesa Muncan, Renée R. C. E. Schreurs, François Jean, Carla M. S. Ribeiro

**Affiliations:** a Department of Experimental Immunology, Amsterdam UMC, University of Amsterdam, Amsterdam, The Netherlands; b Infectious Diseases & Inflammatory Diseases Programs, Amsterdam Institute for Infection & Immunity, Amsterdam, The Netherlands; c Amsterdam Gastroenterology & Metabolism, University of Amsterdam, Amsterdam, The Netherlands; d Department of Microbiology and Immunology, Life Sciences Institute, University of British Columbia, Vancouver, Canada; e Amsterdam Gastroenterology Endocrinology and Metabolism, Tytgat institute for Intestinal and Liver research, Amsterdam UMC, University of Amsterdam, Amsterdam, The Netherlands; f Faculty of Health Sciences, Simon Fraser University, Burnaby, Canada

**Keywords:** Viral entry routes, drug repurposing, extrapulmonary COVID-19, host-directed antiviral therapy, autophagy, Human intestinal organoids, Intestinal barrier function, Intestinal damage

## Abstract

SARS-CoV-2, the causative virus of COVID-19, continues to threaten global public health. COVID-19 is a multi-organ disease, causing not only respiratory distress, but also extrapulmonary manifestations, including gastrointestinal symptoms with SARS-CoV-2 RNA shedding in stool long after respiratory clearance. Despite global vaccination and existing antiviral treatments, variants of concern are still emerging and circulating. Of note, new Omicron BA.5 sublineages both increasingly evade neutralizing antibodies and demonstrate an increased preference for entry via the endocytic entry route. Alternative to direct-acting antivirals, host-directed therapies interfere with host mechanisms hijacked by viruses, and enhance cell-mediated resistance with a reduced likelihood of drug resistance development. Here, we demonstrate that the autophagy-blocking therapeutic berbamine dihydrochloride robustly prevents SARS-CoV-2 acquisition by human intestinal epithelial cells via an autophagy-mediated BNIP3 mechanism. Strikingly, berbamine dihydrochloride exhibited pan-antiviral activity against Omicron subvariants BA.2 and BA.5 at nanomolar potency, providing a proof of concept for the potential for targeting autophagy machinery to thwart infection of current circulating SARS-CoV-2 subvariants. Furthermore, we show that autophagy-blocking therapies limited virus-induced damage to intestinal barrier function, affirming the therapeutic relevance of autophagy manipulation to avert the intestinal permeability associated with acute COVID-19 and post-COVID-19 syndrome. Our findings underscore that SARS-CoV-2 exploits host autophagy machinery for intestinal dissemination and indicate that repurposed autophagy-based antivirals represent a pertinent therapeutic option to boost protection and ameliorate disease pathogenesis against current and future SARS-CoV-2 variants of concern.

## Introduction

Since December 2019, when the Severe Acute Respiratory Syndrome Coronavirus 2 (SARS-CoV-2) was first isolated in Wuhan, China, SARS-CoV-2 has taken the world by storm. The collaborative and international scientific response to the resultant COVID-19 pandemic led to the rapid development of highly effective vaccines that have become the cornerstone of the global public health response to SARS-CoV-2. However, the effectiveness of these vaccines has been steadily threatened by emerging variants of concern (VOCs) that evade humoral responses from infection or vaccination [1–4]. The most recent variant of concern, the Omicron variant, was first detected in November 2021 and has replaced the earlier Alpha and Delta VOCs as the dominant global strain [5]. Currently, Omicron and its subvariants, including BA.5 sublineages, are the predominant SARS-CoV-2 strains circulating worldwide [5]. As with previous VOCs, Omicron subvariants evade antibody responses elicited by vaccines or infection with earlier SARS-CoV-2 variants [4,6]. Additionally, clinically-approved monoclonal antibody treatments have reduced activity against Omicron subvariants BA.1, BA.2, BA.4, BA.5, and currently circulating BQ.1.1 (derived from BA.5), and XBB1.5 (derived from BA.2) [2,3,7,8].

COVID-19 is a multi-organ disease, causing not only respiratory distress and failure, but also a wide range of extrapulmonary symptoms, with up to 17% of patients exhibiting gastrointestinal manifestations, such as nausea and abdominal pain [9,10]. Viral RNA or infectious SARS-CoV-2 can be isolated in stool samples from approximately 50% of COVID-19 patients and remains detectable long after respiratory symptoms subside [11–13]. Furthermore, studies using human intestinal organoid models have reported that SARS-CoV-2 infects the most abundant intestinal cell type, enterocytes, which produce large quantities of infectious SARS-CoV-2, thereby affirming the role of the gastrointestinal tract in virus dissemination and disease pathogenesis [9–11,14,15]. Thus, human pre-clinical studies are paramount to identify strategies to intervene in SARS-CoV-2 intestinal dissemination and lingering impairments associated with post-COVID-19 syndrome. In particular, host-directed therapeutics have great potential to offer increased protection against current and future emerging VOCs, since they are broad-spectrum and relatively impervious to the development of antiviral resistance [16–19]. Here, we aimed to enhance host mucosal immune defences, with a focus on the antiviral role of autophagy pathways, in order to identify host-directed antiviral strategies to prevent SARS-CoV-2 infection.

Macroautophagy/autophagy is an intracellular lysosomal degradation mechanism that ultimately results in the breakdown of intracellular cargo including invading viruses. Specialized double-membrane vesicles coated with the canonical autophagy marker Microtubule-associated proteins 1A/1B light chain 3B (LC3), termed autophagosomes, sequester viruses or viral components. These virus-laden autophagosomes, including cytosolic cargo and host autophagy receptors, such as ubiquitin-binding protein p62 (p62, also known as sequestosome 1), are then targeted for lysosomal degradation. Notably, autophagy not only functions as an antiviral defence mechanism, but also represents a gatekeeper mechanism for intestinal homeostasis [20]. In the intestine, autophagy proteins are required for the secretory function of intestinal epithelial cells (IECs), and dysregulation of autophagy has been implicated in the severity of chronic inflammatory diseases of the bowel such as Crohn’s disease [20]. In IECs, autophagy has been demonstrated to maintain cellular homeostasis and repair the intestine [21,22], supporting its essential role in intestinal barrier function upon cellular stress and infection.

It is widely acknowledged that positive-strand RNA viruses, including SARS-CoV-2, can manipulate autophagy pathways [23–26]. Like other coronaviruses, SARS-CoV-2 stimulates the formation of RNA-replication organelles at double-membrane vesicles (DMVs), which structurally resemble autophagy vesicles and are also LC3+ in the case of mouse hepatitis coronavirus [25,27]. This suggests that SARS-CoV-2 has evolved strategies to hijack acidic autophagy vesicles and membranes to promote their own replication. Furthermore, recent data have also demonstrated that SARS-CoV-2 actively impairs autophagosome-lysosome fusion, thereby limiting the degradative activity of autophagy [23,24,28]. It has been hypothesized that the function of this virus-induced autophagy block is to prevent autophagy-mediated degradation of both virions and lipids that are necessary for DMV formation [25].

Most recently, several peer-reviewed studies [6,29,30] have demonstrated that although SARS-CoV-2 Omicron subvariants can use both the cell-surface routing and endosomal entry routes, BA.1 and BA.2 subvariants in particular, and to a lesser extent BA.5, preferentially utilize the latter in VeroE6, HEK-293T, and Calu3 human cell lines [6,29,30]. The endosomal virus entry route relies on an acidic intravesicular environment and cleavage by endosomal cathepsins to drive fusion with host intracellular membranes and escape into the cytoplasm, unlike the classical cell-surface Transmembrane protease serine 2 (TMPRSS2)-mediated entry route favoured by the ancestral Wuhan-Hu-1 strain and the earlier Delta VOCs [6,29]. Notably, classical experimental inhibitors of autophagosome-lysosome fusion (i.e. inhibitors of autophagy flux) bafilomycin A1 and chloroquine potently block the entry of both Omicron BA.1 and Delta SARS-CoV-2 variants *in vitro*, suggesting a particularly key role for autophagy pathways in supporting the replication of Omicron subvariants [29,30].

Pharmaceutical modulation of autophagy is already utilized for the treatment of non-infectious conditions, such as cancer [31], epilepsy [32], and neuroinflammation [33]. Recently, we have demonstrated the potential for clinically-approved autophagy-modulating drugs to limit intestinal HIV-1 acquisition and suppress ongoing HIV-1 replication in diverse cellular targets in human *ex vivo* tissue infection models, underlining host autophagy as a relevant target for antiviral therapies [17,34]. The intimate interplay between autophagy and endocytosis pathways in viral entry routes [35], alongside the manipulation of autophagy by SARS-CoV-2 [23–26], prompted us to investigate the potential of autophagy-targeting therapeutics to combat SARS-CoV-2 dissemination.

Here, we show that SARS-CoV-2 utilizes the endosomal/autophagy entry route to infect intestinal epithelial cells, which is accompanied by loss of intestinal integrity. Notably, our data shows that a selection of autophagy-blocking drugs can suppress intestinal SARS-CoV-2 infection as well as prevent SARS-CoV-2-mediated disruption of the intestinal barrier. These findings underscore the therapeutic relevance of targeting host autophagy, and in particular autophagy mechanisms mediated by the host BCL2-Interacting protein 3 (BNIP3), to intervene in infections with emerging SARS-CoV-2 VOCs.

## Materials and methods

### Ethics statement

Fetal material was used as determined by Dutch law (Wet Foetal Weefsel), which states that human foetal tissues/cells can only be used for medical purposes, medical and scientific research, and medical and scientific education. Human foetal intestinal tissue samples (gestational age 17–18 weeks) were obtained from the HIS Mouse Facility of the Academic Medical Center, Amsterdam. All material has been collected from donors from whom written informed consent has been obtained for the use of the material for research purposes. The foetal donor information is anonymized and is not available to the Amsterdam UMC.

### Intestinal epithelial monolayers

Human fetal intestinal organoids, for which generation and culture conditions are described in in supplementary materials M1, were used to generate human intestinal monolayers as described previously [36]. Briefly, 3.0 µm pore 24-well transwell inserts (Corning) were coated with 20 µg/mL rat collagen type 1 (Ibidi) in 0.1% acetic acid (Sigma Aldrich). Single cells were obtained from gut organoids using TrypLE (Gibco, Thermo Fischer Scientific) digestion and suspended in expansion medium – Intesticult human organoid growth medium (OGM; STEMCELL Technologies) components “Human Basal Medium” and “Organoid Supplement” in a 1:1 ratio supplemented with penicillin/streptomycin (10 U/ml and 10 μg/ml, respectively; Thermo Fisher Scientific) - supplemented with Y-27632 (STEMCELL Technologies), followed by seeding onto the collagen coated transwell inserts. For the first 7 days the monolayers were cultured in expansion medium. From day 7 onwards all monolayers were cultured in a differentiation medium (1:1 mixture of OGM component “Human Basal Medium” and Advanced DMEM/F12; (Thermo Fisher Scientific)) supplemented with 10 mM GlutaMAX (Thermo Fisher Scientific), 10 mM HEPES (Sigma), and 1X Penicillin/Streptomycin (Thermo Fisher Scientific), and refreshed every 3–4 days. The integrity of the monolayer intestinal barrier was determined by transepithelial electrical resistance (TEER) measurement using an Epithelial Volt/Ohm meter (EVOM2). TEER values (Ω.cm^2^) were calculated as described in [37]. Monolayers were deemed sufficiently formed when the TEER was over 200 Ω.cm^2^. The permeability of the intestinal epithelial monolayer was assessed using FITC-conjugated dextran (4 kDa FD4, Sigma Aldrich) translocation assay from the apical to the basolateral compartment as described in supplementary materials M2. ACE2 expression in intestinal tissues was determined by fluorescence microscopy and flow cytometry analyses as described in supplementary materials M3.

### Cell lines

The Caco-2 cell line used in pseudovirus infection assays were a gift from Dr. Marta Bermejo Jambrina, Amsterdam UMC. The U87 cell line stably expressing CD4 and wild-type CCR5 co-receptor was obtained through the NIH AIDS Reagent Program, Division of AIDS, NIAID, NIH: U87 CD4^+^CCR5^+^ cells, ARP-4035, contributed by Drs. HongKui Deng and Dan Littman [17,34,38]. Autophagy reporter cells were generated via retroviral transduction of U87.CD4.CCR5 with pBABE-mCherry-GFP-LC3 (Addgene 22418; gift from Prof. Jayanta Debnath [39]), as described previously [17,40], hereafter referred to as U87.LC3-mCherry-GFP cells. Culture conditions are described in supplementary materials M4.

### Autophagy-modulating compounds

Compounds utilized in this study to modulate autophagy were: berbamine dihydrochloride (Sigma 547190, “BBM”), Daurisoline (Sigma SML0597-5MG, “DAS”), and Bafilomycin A1 (Invivogen tlrl-baf1). DAS and Bafilomycin A1 stock were resuspended in sterile DMSO, and BBM in sterile water. Prior to the treatment of cell lines or primary cell culture, stocks were further diluted in cell culture medium. Cell viability upon drug treatment was measured as described in Supplementary materials M5.

### Autophagy flux

U87.LC3-mCherry-GFP cells were incubated with BBM (73.4 μM), DAS (60 μM), Bafilomycin A1 control (200 nM), or left untreated, for 24 h. GFP and mCherry fluorescence was measured by multiparameter (FACS LSR-FORTESSA, BD Biosciences) and imaging flow cytometry analysis (ImageStream Mk II, Amnis) as previously described [40,41]. Reduction of GFP signal is representative of autophagy flux and was determined in live, singlet, mCherry^+^GFP^+^ U87.LC3-mCherry-GFP cells in untreated and drug-treated conditions. The control gate to determine autophagy flux was set in cells treated with bafilomycin A1 (200 nM) [17,40,41].

### Intracellular p62 accumulation

Intracellular p62 accumulation was measured in Caco-2 cells treated with BBM (36.7 μM), or DAS (30 μM), or left untreated, for 72 h. Cells fixed in 4% paraformaldehyde (PFA; Electron Microscopy Sciences) were permeabilized with PBS containing 0.5% saponin and 1% bovine serum albumin (BSA; both Sigma), followed by staining with either mouse anti-p62 (IgG2a, 5 μg/mL, Abcam ab56416) or mouse IgG2a isotype control (5 μg/mL, eBioscience 14-4724-85), and thereafter staining with goat anti-mouse IgG2a-Alexa Fluor 488 (Invitrogen A11029). Intracellular p62 accumulation was quantified using flow cytometry (FACSCanto II, BD Biosciences).

### SARS-CoV-2 pseudovirus infection assays

Single-round infection pseudotyped SARS-CoV-2 (NL4.3-ΔENV-luc-GB-pseudo-SARS-CoV-2-SpikeΔ21) was generated as described previously [42,43] and in Supplementary material M6. The SARS-CoV-2 spike plasmid HDM-IDTSpike-fixK-Δ21 was obtained through BEI Resources, NIAID, NIH: Vector pHDM Containing the SARS-Related Coronavirus 2 Spike Glycoprotein Gene, D614G Mutant with C-Terminal Deletion, NR-53765. The adjusted HIV-1 backbone plasmid (pNL4-3.Luc.R-S-) containing previously described stabilizing mutations in the capsid protein (PMID:12547912) and a firefly luciferase gene in the *nef* open reading frame was provided by Dr. N.A. Kootstra (Amsterdam UMC).

Caco-2 cells were cultured to approximately 80% confluency, or intestinal epithelial monolayers were cultured to TEER >200 Ω.cm^2^ prior to overnight treatment with BBM (Caco-2: 14.7, 7.3, 3.7 μM, or 1.8 μM; organoid monolayers: 36.7 μM), DAS (Caco-2: 60, 30, 15 μM, or 7.5 μM; organoid monolayers: 30 μM), α-ACE2-antibody (R&D AF933) or isotype control (R&D AB-108-c), camostat mesylate (Sigma SML0057) or left untreated. Caco-2 or intestinal epithelial monolayers were then infected with 87.5–350 plaque forming units (PFU) of NL4.3-ΔENV-luc-GB-pseudo-SARS-CoV-2-SpikeΔ21. Luciferase activity was measured using a luciferase assay system (Britelite Plus Kit, Perkin Elmer cat #6066761) according to the manufacturer’s instructions.

### Confocal microscopy

Intestinal epithelial monolayers were fixed in 4% paraformaldehyde (PFA; Electron Microscopy Sciences) for at least 30 min at room temperature (RT), extensively washed with PBS. Membranes were then cut out of the insert and permeabilized with 0.5% Triton-X100 for 10 min, and subsequently blocked with PBS containing 3% BSA with 0,5% Tween-20 for 1 h at RT. Cells were incubated overnight at 4°C with primary antibody Mouse IgG1 anti-p24 (clone KC57, Beckman Coulter) diluted 1:100 in PBS containing 3% BSA with 0,5% Tween-20, followed by secondary antibody Goat anti-Mouse IgG1 Alexa 647 diluted at 1:400 and the probe Phalloidin CruzFluor^TM^-488 (Santa Cruz Biotechnology) diluted at 1:1500 in PBS containing 3% BSA with 0,5% Tween-20 for 1 h at RT. Nuclei were stained with 300nM DAPI (Invitrogen). Cells were subsequently mounted in Prolong Gold Diamond Antifade mountant (Invitrogen). Samples were imaged on a Leica TCSS SP8 X mounted on a Leica DMI6000 and analysed using LAS X. Quantitative analysis of fluorescence intensity were performed by measuring the mean grey value with ImageJ software.

### RNA interference

RNA interference was performed using the Neon Transfection System according to manufacturer’s protocol (Thermo Fisher). Caco-2 cells were transfected with different short interfering (si) SMARTpool RNAs (Dharmacon), namely siSNAP29 (M-011935-00), or siBNIP3 (M-004636-01), or siNon-Target as a control (D-001206-13) at end concentrations of 0.1 µM siRNA per million of cells. Transfected cells, as well as non-transfected Caco-2 cells as an additional control, were seeded in 24-well plates in EMEM (ATCC #30-2003) with 8% FCS (Bio-connect), 2 mM L-glutamine (Lonza), and 1x non-essential amino acids (SanBio SCC0823), without antibiotics. After 32 h, adherent cells were washed and incubated overnight either with optimized concentrations of BBM (3.7 μM) or left untreated. Silencing of expression of target proteins in Caco-2 cells was confirmed by quantitative real-time PCR (Figure 5C, Supplementary Figure 1 or Supplementary Table 1).

### SARS-CoV-2 Omicron BA.2 and BA.5 infection assays

SARS-CoV-2 Omicron BA.2 or BA.5 stocks were produced as described in supplementary materials M7 and diluted in cell-specific media to a multiplicity of infection (MOI) of 2. Caco-2 cells were seeded at a concentration of 8,000 cells/well in 96-well plates the day before infection. Cells were pre-treated with compounds and then incubated with the virus, followed by fixation of the cells with 4% formalin in PBS for 30 min. Cells were subsequently washed with PBS, permeabilized with 0.1% Triton X-100 for 10 min and blocked with 1% BSA in PBS for 1 h at RT, followed by immunostaining with the mouse primary dsRNA antibody (J2-1904, Scicons English and Scientific Consulting) and rabbit primary SARS-CoV-2 antibody (HL344, Genetex GTX635679) at working dilutions of 1:1000 in 1% BSA in PBS overnight at 4°C. Secondary antibodies were used at a 1:2000 dilution in 1% BSA in PBS and included the goat anti-mouse IgG Alexa Fluor 488 (A11001, Invitrogen) and goat anti-rabbit IgG Alexa Fluor 555 (A21428, Invitrogen) with the nuclear stain Hoechst 33342 at 1.5 µg/mL for 1 h at RT. After washing with PBS, the plates were imaged on a high-content screening (HCS) platform (CellInsight CX7 HCS, Thermo Fisher Scientific) with a 10X objective.

### High-content screening of SARS-CoV-2 infection

Monitoring of the total number of cells (based on nuclei staining) and the number of virus-infected cells (based on dsRNA or nucleocapsid staining) was performed using the CellInsight CX7 HCS platform (Thermo Fisher Scientific), as previously described [44,45]. Briefly, nuclei are identified and counted using the 350⁄461 nm wavelength (Hoechst 33342); cell debris and other particles are removed based on a size filter tool. A region of interest (ROI, or “circle”) is then drawn around each host cell and validated against the bright field image to correspond with host cell membranes. The ROI encompasses the “spots” where dsRNA (485/521 nm wavelength) or SARS-CoV-2 nucleocapsid (549/600 nm wavelength) are localized. Finally, the software (HCS Studio Cell Analysis Software, version 4.0) identifies, counts, and measures the pixel area and intensity of the “spots” within the “circle.” The fluorescence measured within each cell (circle) is then added and quantified for each well. The total circle-spot intensity of each well corresponds to intracellular virus levels (Z’ > 0.7) and is normalized to noninfected cells and to infected cells with 1% DMSO. Nine fields were sampled from each well. Nuclei stain (Hoechst 33342) was also used to quantify cell loss (due to cytotoxicity or loss of adherence) and to verify that the changes in viral infection did not result from a decrease in cell numbers.

### Median effective dose (EC_50_) curves

Intracellular dose response (EC_50_ values) for BBM and camostat mesylate against SARS-CoV-2 Omicron BA.2 and BA.5 was determined by pre-treating Caco-2 cells with serially diluted compound, followed by virus infection. Viral infection was detected by staining for dsRNA or nucleocapsid signal and quantified as described above. EC_50_ experiments were performed with two technical replicates in each experiment and EC_50_ experiments were repeated at least three times. Intracellular fluorescent levels were interpolated to a negative control (0.1% DMSO, no infection) = 0, and positive control (0.1% DMSO, with infection) = 100.

### Statistical analyses

Data were analyzed using FlowJo, LLC, version 10 (Treestar), IDEAS version 6.3 (Amnis), and/or GraphPad Prism 9™ (GraphPad Software, Inc.). A two-tailed, parametric Student's *t-*test was performed for paired observations, and a two-tailed, unpaired *t-*test was performed for independent observations. For data shown in relative, data were normalized to untreated, virus infected samples (set at 1). A one-sample *t-*test was then utilized to compare fold changes in experimental conditions (drug-treated-virus infected samples) to the hypothetical population mean of 1, in line with [17]. The GraphPad Prism 9 nonlinear regression fit modelling variable slope was used to generate a dose–response curve for EC_50_ values, [Y = Bottom + (Top–Bottom)/(1 + 10^((LogIC_50_-X)*HillSlope))], constrained to top = 100, bottom = 0, as in [18]. Statistical analyses were performed using GraphPad Prism 9 and significance was set at *P < 0.05, **P < 0.01, ***P < 0.001.

## Results

### SARS-CoV-2 infection of human intestinal epithelial monolayers is accompanied by loss of intestinal barrier integrity

**Figure 1.**
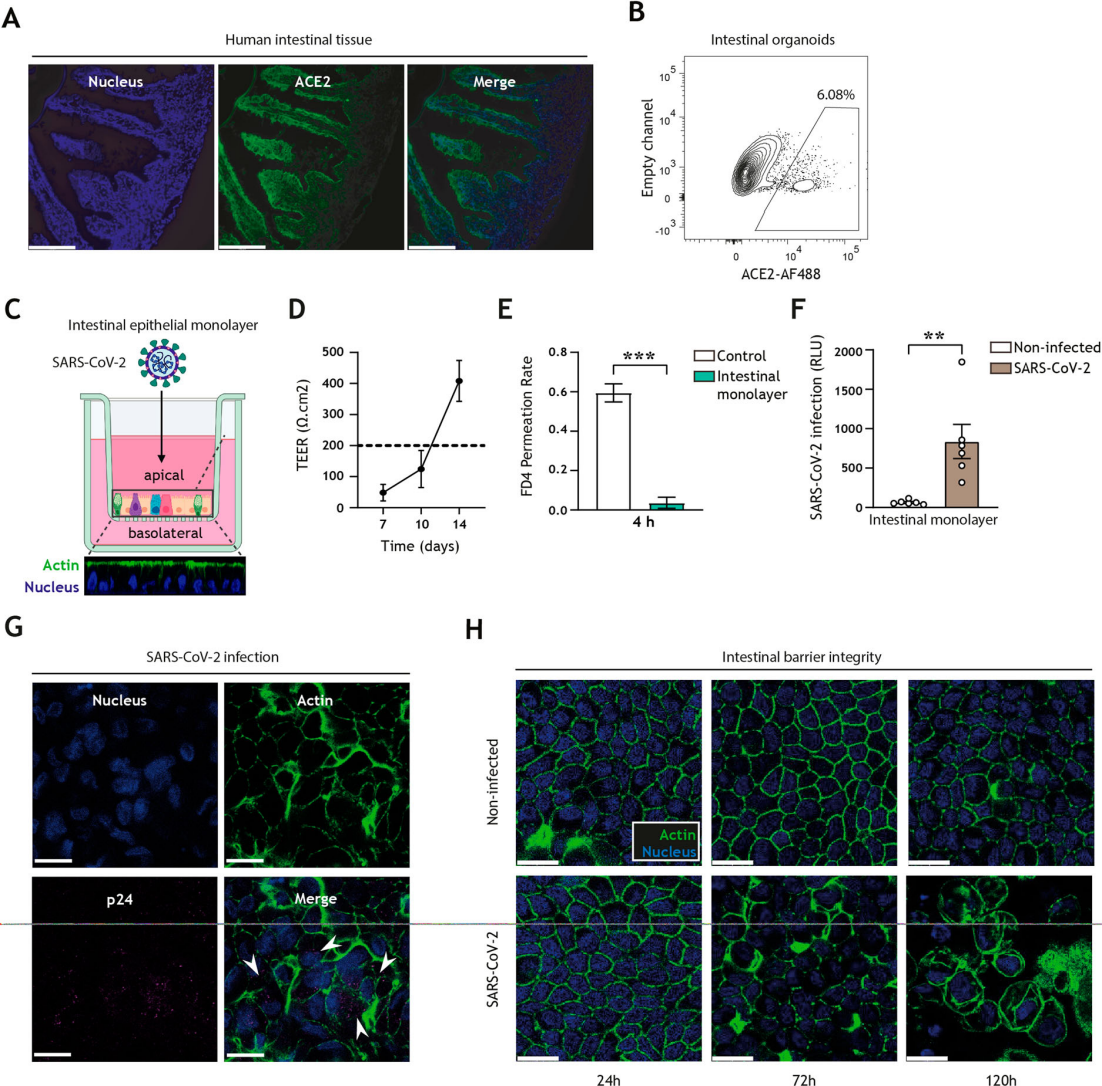
SARS-CoV-2 pseudovirus infects human intestinal monolayers and disrupts intestinal barrier integrity. (A) ACE2 expression (green) in human intestinal tissue, determined by fluorescence microscopy. Nuclei (DAPI) are shown in blue. Scale bar = 166 micron. Representative of *n *= 2. (B) ACE2-positive cells in 3D organoid cultures derived from human intestinal tissue, determined by flow cytometry. Representative of *n *= 3. (C) Graphical representation of the intestinal organoid monolayer infection model, including representative confocal images of a top view and side view of the monolayer resting on a 3.0 micron pore 24-well transwell insert. Actin (Phalloidin) is shown in green and nuclei (DAPI) in blue. (D) TEER values of intestinal epithelial monolayers were measured at 7, 10, and 14 days of culture. Broken line indicates TEER value of 200 Ω.cm^2^, *n *= 6 donors. (E) FD4 permeability of intestinal epithelial monolayers cultured for 14 days and measured at 4 h post-FD4 addition in *n *= 4 donors. Permeability is expressed as FD4 permeation rate: FD4 basolateral^t=4^(μg)/FD4 apical^t=0^(μg) (see supplementary material M2). Controls are transwell membranes without monolayers. ***P < 0.001, Unpaired *t*-test. (F) Viral infection of intestinal epithelial monolayers, determined by luciferase activity (Relative light units, RLU). Intestinal epithelial monolayers cultured to TEER >200 Ω.cm^2^ prior to exposure to SARS-CoV-2 pseudovirus for 5 days. Open circles represent individual donors, *n *= 6 donors. **P < 0.0052, unpaired *t-*test. (G) SARS-CoV-2 infection of intestinal epithelial monolayers, determined by confocal imaging. SARS-CoV-2 pseudovirus particles (Mouse IgG1 anti-p24) are shown in magenta, actin (Phalloidin) is shown in green and nuclei (DAPI) in blue 5 days post-inoculation. Scale bar = 15 micron, representative of *n *= 3. (H) Changes in morphology of intestinal epithelial monolayers upon infection for 24, 72, or 120 h with SARS-CoV-2 pseudovirus, determined by confocal microscopy. Actin (Phalloidin) is shown in green and nuclei (DAPI) in blue. Scale bar = 15 micron, representative of *n *= 2 donors.

We first confirmed that the obligate entry receptor for SARS-CoV-2 entry, ACE2, is expressed in both human intestinal tissues as well as in human-derived organoid models (Figure 1(A,B)). To monitor intestinal SARS-CoV-2 entry, we utilized our intestinal epithelial monolayer model [36,46], which in contrast to 3D intestinal organoid models readily permits access to the luminal (apical) and mucosal (basolateral) compartments, and thereby allows administration of viruses and antiviral drugs without the need for microinjection and in a manner that closely recapitulates *in vivo* intestinal architecture (Figure 1(C)). The integrity and paracellular permeability of intestinal epithelial monolayers were routinely monitored by transepithelial electrical resistance (TEER) measurement (with > 200 Ω.cm^2^ indicative of a confluent monolayer; Figure 1(D)) and FITC-dextran permeation rate (Figure 1(E)), respectively. Permissiveness of intestinal epithelial monolayers to SARS-CoV-2 pseudovirus bearing the ancestral spike protein was validated by luminescence measurement (Figure 1(F)) as well as confocal imaging (Figure 1(G)), showing that this human intestinal epithelial monolayer model is suitable to monitor human intestinal SARS-CoV-2 acquisition. Notably, entry of SARS-CoV-2 into intestinal epithelial cells resulted in disruption of monolayer barrier integrity 72 h post-inoculation, as evident by distinct morphological changes of the intestinal epithelial cells and loss of cell–cell contact, which were increasingly exacerbated over time (Figure 1(H)).

### SARS-CoV-2 hijacks the intracellular autophagy pathway to establish infection of human intestinal epithelial cells

**Figure 2.**
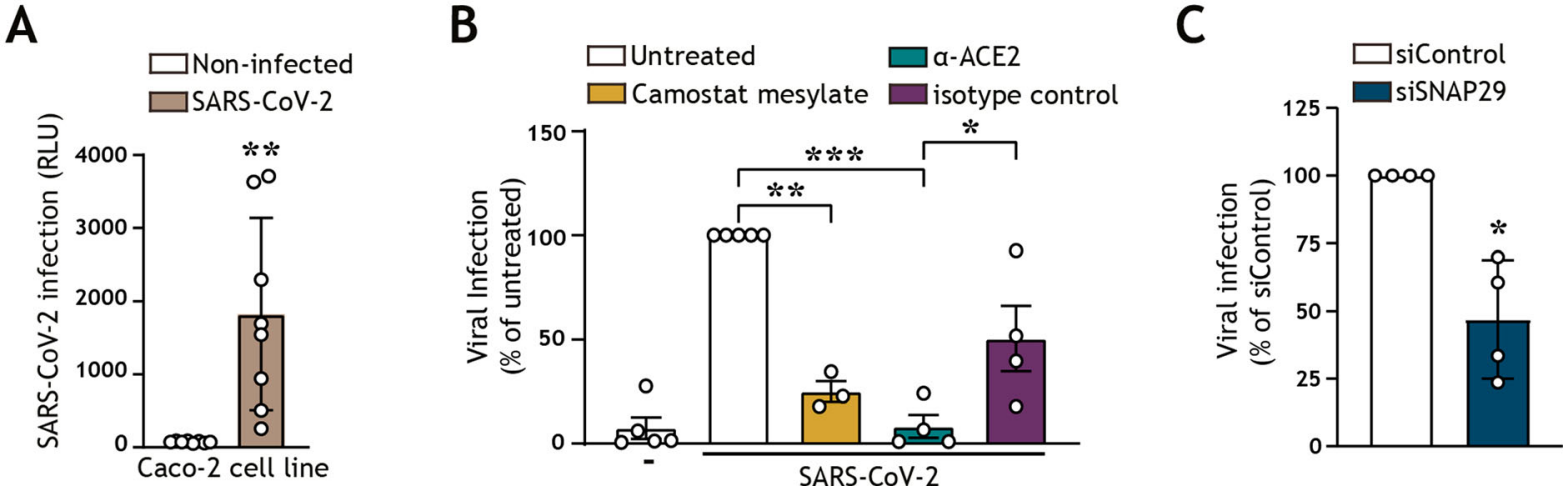
SARS-CoV-2 pseudovirus utilizes both the cell-surface entry route as well as the autophagy intracellular pathway to infect intestinal epithelial cells (A) Viral infection of Caco-2 cell line, determined by luciferase activity (RLU). Caco-2 cells were cultured to approximately 80% confluency, prior to infection with SARS-CoV-2 pseudovirus for 72 h. Open circles represent individual replicates, *n *= 8. **P < 0.05, unpaired. (B) Viral infection of Caco-2 cells pre-treated for 16 h with α-ACE2-antibody or isotype control, camostat mesylate, or left untreated, and subsequently exposed to SARS-CoV-2 pseudovirus for 72 h, determined by luciferase activity (RLU). Open circles represent individual replicates, *n *= 3–4, and data were normalized to the infected but untreated control. **P < 0.01, ***P < 0.001, One-sample *t-*test. α-ACE2-antibody versus isotype control *P < 0.044, Unpaired *t-*test. (C) Viral infection of Caco-2 cells upon transfection with control siRNA, or siSNAP29 followed by exposure to SARS-CoV-2 pseudovirus for 72 h, determined by luciferase activity (RLU). Open circles represent individual replicates, *n *= 4, and data were normalized to the infected siRNA treated control. *P < 0.05, One-sample *t-*test.

In concordance with other reports, we additionally showed that SARS-CoV-2 entry could be detected in the Caco-2 human colon cancer epithelial cell line (Figure 2(A)). We validated that a block of the ACE2 surface receptor or inhibition of TMPRSS2 surface serine protease via camostat mesylate decreased viral entry into epithelial cells, underpinning the ability of SARS-CoV-2 to utilize the TMPRSS2-dependent cell-surface entry route to establish infection of intestinal epithelial cells (Figure 2(B)). Autophagy is a regulator of intestinal homeostasis and functions as an antiviral defence mechanism by mediating lysosomal viral degradation [17,20,34]. To investigate the role of autophagy flux on SARS-CoV-2 entry of epithelial cells, we employed RNA interference (RNAi) technology to knock down the expression of autophagy molecule Synaptosomal-associated protein 29 (SNAP29), which is an adaptor molecule that links autophagosome-associated syntaxin 17 (STX17) with lysosomal vesicle-associated membrane protein 8 (VAMP8), thereby driving autophagosome-lysosome fusion [47]. Notably, the knockdown of SNAP29, which leads to a block in autophagy flux and reduced levels of acidic autophagy vesicles, resulted in decreased SARS-CoV-2 infection, suggesting the ability of SARS-CoV-2 to utilize not only the TMPRSS2-mediated cell-surface route, but an additional autophagy/endosomal route for viral entry in intestinal epithelial cells (Figures 2(B,C), Supplementary view figure 1, Supplementary view table 1). Taken together, these data demonstrate that SARS-CoV-2 readily infects human intestinal epithelial cells as well as compromises the intestinal barrier function. Furthermore, we have demonstrated that SARS-CoV-2 hijacks autophagy mechanisms to establish intestinal infection.

### Autophagy-blocking therapeutics suppress intestinal SARS-CoV-2 acquisition and restore intestinal barrier integrity

**Figure 3.**
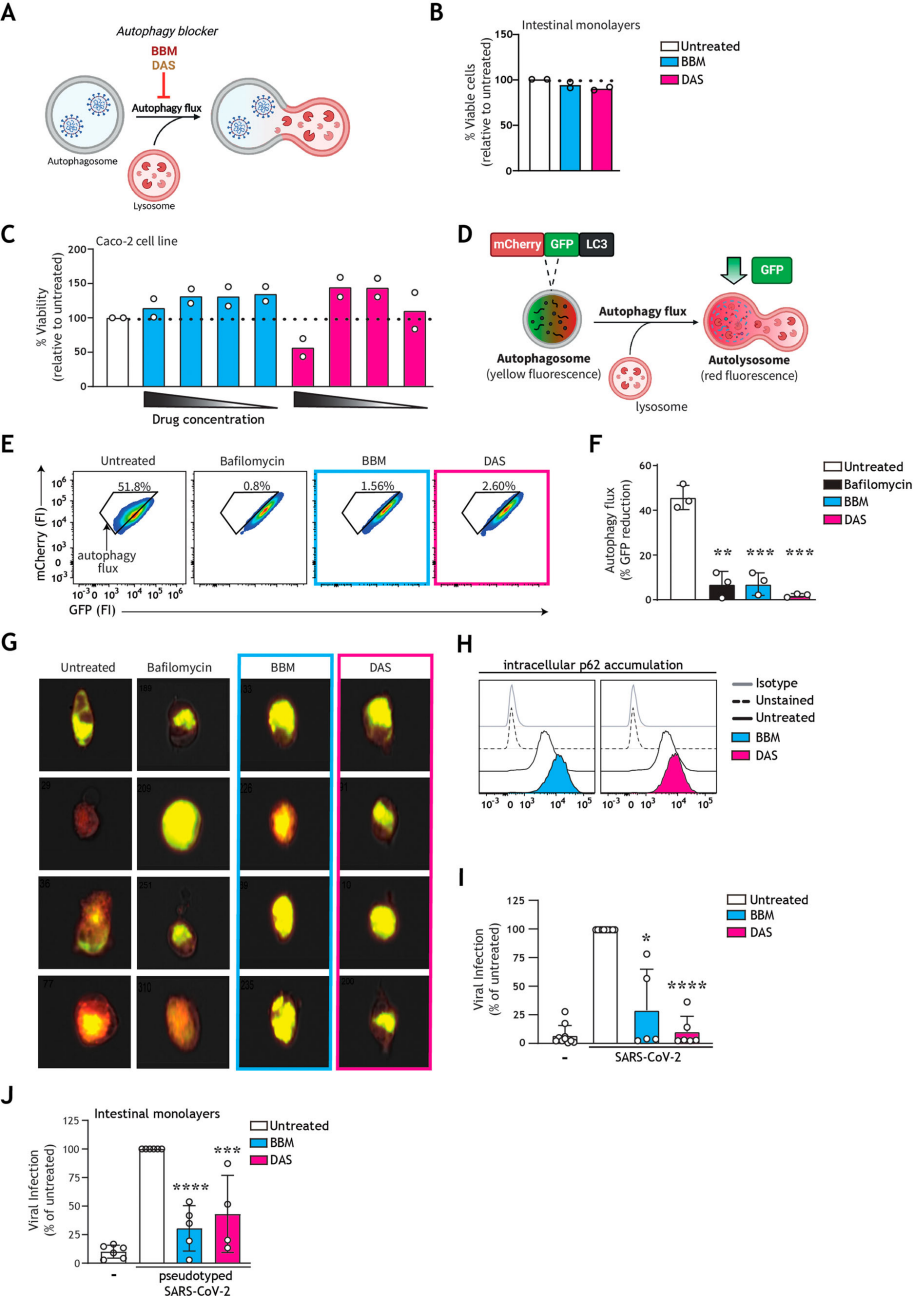
Pharmacological modulation of autophagy prevents SARS-CoV-2 pseudovirus acquisition by intestinal epithelial cells. (A) Graphical representation of the modes of action by which BBM and DAS modulate the autophagy pathway. (B, C) % Cell viability upon treatment of intestinal monolayers (B) or Caco-2 cell line (C) with BBM or DAS, determined by ATP-based CellTiter-Glo assay. (B) Intestinal epithelial monolayers were treated with optimized concentrations of BBM, or DAS, or left untreated, for 60 h. Open circles represent individual donors, *n *= 2 donors. (C) Caco-2 cells were treated with serial dilutions of BBM, or DAS, or left untreated, for 72 h. Open circles represent individual replicates, *n *= 2. (D) Graphical representation of the U87.LC3-mCherry-GFP autophagy reporter cell line employed to monitor autophagy flux. The GFP tag in the U87.LC3-mCherry-GFP cells are acid-sensitive and are quenched upon autophagosome acidification, permitting the monitoring of autophagy flux by measuring reduction in GFP signal. (E, F, G) Autophagy flux in U87.LC3-mCherry-GFP cells upon incubation with Bafilomycin A1 control, BBM, DAS, or left untreated, for 24 h. Autophagy flux was monitored by % GFP signal reduction, determined by flow cytometry (E, F) and imaging flow cytometry (G). Representative flow cytometry plots (E) and quantification of flow cytometry data (F), data are mean ± SE of *n* = 3 replicates represented by open circles; **P < 0.01, ***P < 0.001, Student's *t-*test. (G) Representative imaging flow cytometry overlays of bright field, mCherry, and GFP signal, four individual cells shown per condition are representative of *n* = 3 replicates; (H) Intracellular p62 accumulation in Caco-2 cell line upon treatment with BBM, DAS, or left untreated, for 72 h, measured by flow cytometer. Data are representative flow cytometry plots of *n* = 2 independent experiments performed in triplicate. (I, J) Viral infection of Caco-2 cell line (I) or intestinal monolayers (J) pre-treated with BBM, DAS, or left untreated, determined by luciferase activity (RLU). (I) Caco-2 cells were infected with SARS-CoV-2 pseudovirus for 72 h, data are mean ± SE of *n* = 6 replicates represented by open circles; *P = 0.0115, ****P < 0.0001, One sample *t*-test. (J) Intestinal epithelial monolayers were infected with SARS-CoV-2 pseudovirus for 5 days. Open circles represent individual replicates, *n *= 4 intestinal donors, ***P = 0.0005, ****P < 0.0001, one-sample *t-*test.

Next, we have therefore investigated the impact of autophagy-modulating strategies to intervene in intestinal SARS-CoV-2 infection and associated gut pathogenesis. To this end, we have screened a panel of ten pre-clinical or clinically approved autophagy-blocking/inhibiting small molecules, based on the hypothesis that autophagy blockers could prevent SARS-CoV-2 interaction with cathepsins within acidic autophagy vesicles to impair SARS-CoV-2 entry via the endosomal route. The panel comprised of drugs targeting autophagosome-lysosome fusion or vesicle acidification in varying manners, such as disrupting proton gradient, blocking sodium-potassium ATPase function, or preventing fusion of lysosomes with other vesicles. Eight drugs did not pass a screening for robust impact on autophagy at non-toxic concentrations in human cells, thus we selected two drugs that were non-toxic in human intestinal organoid models: berbamine dihydrochloride (hereafter BBM) [47], and daurisoline (hereafter DAS) [48] (Figure 3(A,B)).

Optimized non-toxic concentrations of BBM and DAS were established in both primary human intestinal monolayers (Figure 3(B)) and Caco-2 cells (Figure 3(C)), via an ATP-based cell viability assay. Intracellular ATP levels are a determinant of metabolic activity and thereby used as an indicator of cell viability [49]. The slight increase of viability specifically in Caco-2 cell line upon drug treatment may stem from altered metabolic activity following autophagy modulation [50]. Next, the capacity of these drugs to regulate autophagy flux (i.e. autophagy-mediated degradation via the lysosome) was tested on the engineered U87 human cell line by both flow cytometer and imaging flow cytometry as previously described [17,41]. Briefly, in the standardized U87.LC3-mCherry-GFP autophagy reporter system (Figure 3(D)), the canonical autophagy LC3 molecule is GFP- and mCherry-positive. Reduction in GFP fluorescence represents enhanced autophagy flux [40]. Contrastingly, a block in autophagy flux, as occurs upon treatment with the canonical autophagosome-lysosome fusion experimental inhibitor bafilomycin A1, is represented by a high incidence of double-GFP^+^mCherry^+^ vesicles as evident by increased levels of yellow fluorescent autophagy vesicles (Figure 3(E–G)) [41]. Treatment with BBM or DAS resulted in increased accumulation of yellow-fluorescent autophagosomes to a degree similar to that of the bafilomycin A1 control, representative of arrested autophagy flux (Figure 3(E–G)).

Inhibition of autophagy flux was additionally confirmed in Caco-2 cells by intracellular p62 expression. Similarly, to the autophagosome-bound LC3 molecule, the autophagy receptor p62 associated with the inner leaflet of the autophagosome is degraded together with cargo in the autolysosome. Accumulation of intracellular p62 is thereby representative of a block in autophagic flux [41]. Treatment with BBM or DAS enhanced p62 accumulation, as compared to untreated controls, further confirming the impact of our selection of drugs on autophagy flux. (Figure 3(H)). Next, we aimed to investigate if these autophagy-blocking drugs could also intervene in intestinal SARS-CoV-2 infection. To this end, human intestinal epithelial cells were pre-incubated with optimized concentrations of BBM or DAS and then subsequently infected with SARS-CoV-2 pseudovirus. Notably, treatment with BBM or DAS robustly inhibited SARS-CoV-2 entry of Caco-2 cells (Figure 3(I)), as well as in primary human intestinal epithelial monolayers (Figure 3(J)).

**Figure 4.**
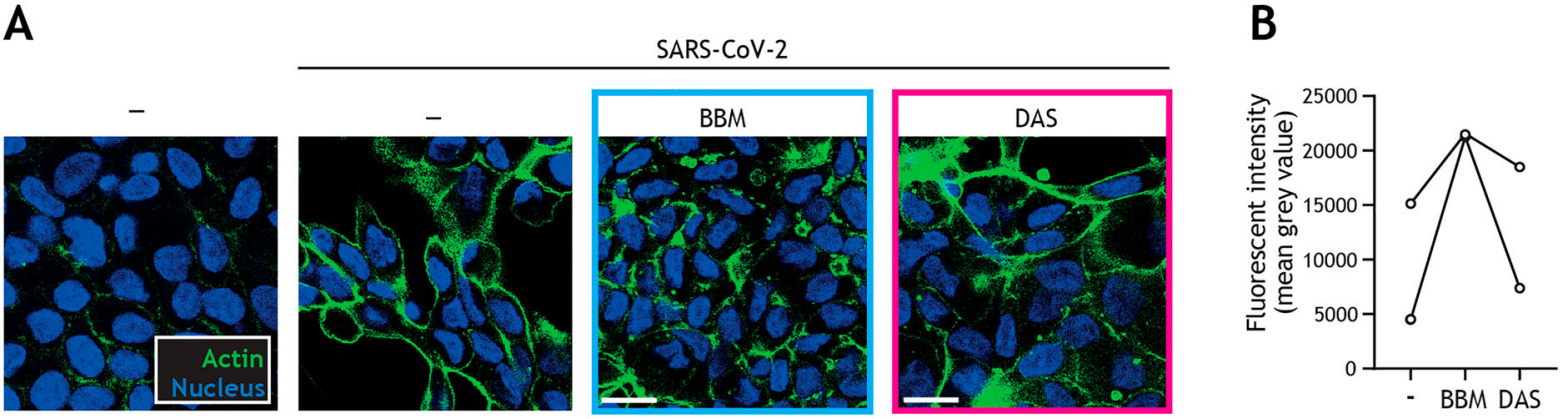
Berbamine limits SARS-CoV-2-mediated impairment of intestinal integrity. (A) Changes in morphology of intestinal epithelial monolayers upon pre-treatment with BBM, DAS, or left untreated for 24 h, followed by exposure to SARS-CoV-2 pseudovirus for 5 days, determined by confocal microscopy. Actin (Phalloidin) is shown in green and nuclei (DAPI) in blue. Scale bar = 15 micron, representative of *n *= 2. (B) Quantitative analysis of fluorescence intensity (FI) of actin staining of intestinal epithelial monolayers as shown in (A). Analyses of fluorescence intensity were performed at original magnification by measuring mean grey value with ImageJ software. Open circles represent averages derived from 5 different fields of view, *n *= 2 intestinal donors.

We next assessed if the same autophagy-blocking drugs could also prevent SARS-CoV-2-induced damage to intestinal barrier function. Strikingly, using cytoskeletal actin and nuclei immunofluorescent staining, we observed that SARS-CoV-2-induced morphological changes of the intestinal epithelial cells and loss of cell–cell contact was largely restored by autophagy blocker BBM, and to a lesser extent by autophagy blocker DAS (Figure 4(A, B)).

### Berbamine exhibits pan-antiviral activity against Omicron subvariants in a BNIP3-dependent manner

**Figure 5.**
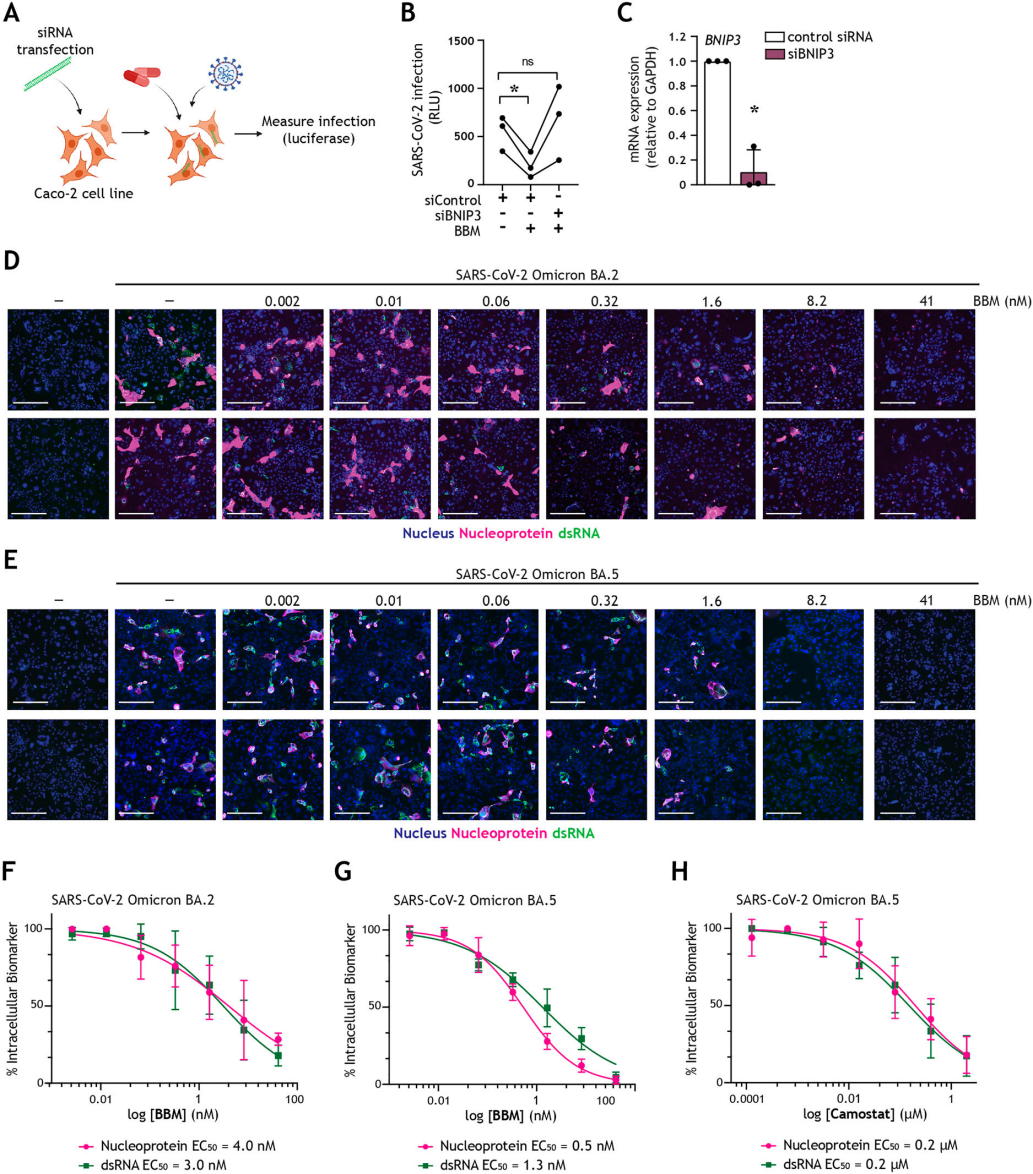
Berbamine abrogates intestinal SARS-CoV-2 replication in a BNIP3-dependent manner (A) Graphical representation of the experimental protocol utilized for viral infection of Caco-2 cells upon transfection with control siRNA or siBNIP3 followed by treatment with BBM as presented in (B, C). (B) Viral infection of Caco-2 cells upon transfection with control siRNA or siBNIP3 followed by treatment with BBM and subsequently exposed to SARS-CoV-2 pseudovirus for 72 h, determined by luciferase activity (RLU). Circles represent individual replicates, *n *= 3; *P < 0.05, Student’s *t-*test. (C) BNIP3 silencing efficiency was determined by real-time PCR. mRNA expression was normalized to GAPDH and set at 1 in cells treated with control-siRNA. *P < 0.05, one-sample *t-*test. (D-H) Viral infection of Caco-2 cells pre-treated with serially diluted BBM for 3 h followed by exposure to SARS-CoV-2 Omicron BA.2 (D, F) or SARS-CoV-2 Omicron BA.5 (E, G) for 48 h. (D, E) Representative fluorescent images of Caco-2 cells treated with the indicated concentrations of BBM. Control images of uninfected and untreated samples are also shown. Hoechst is shown in blue, nucleocapsid in red, and dsRNA in green. Scale bar = 100 micron. (F-H) Dose-response curves were generated for BBM (F, G) or camostat mesylate (H) in Caco-2 cells infected with SARS-CoV-2 Omicron BA.2 (F) or SARS-CoV-2 Omicron BA.5 (H, G), using nucleocapsid (magenta circle) and dsRNA (green square) as infection markers. EC_50_ values were determined using nonlinear regression analysis. Symbols indicate the mean values of *n *= 2 independent experiments.

We then set out to investigate the mechanism underlying the antiviral effect observed upon pharmaceutically introducing a block in autophagy flux. BBM was selected as a representative autophagy-modulating host-directed therapeutic due to its concomitant effective inhibition of SARS-CoV-2 infection and a superior capacity to restore intestinal barrier function, as well as its previously reported anti-tumour and anti-inflammatory effects on human intestinal diseases [31,51,52]. To this end, the expression of BNIP3, a key host molecule involved in controlling autophagy flux by blocking SNAP29-VAMP8 interaction and thereby autophagosome-lysosome fusion [47,53], was silenced in Caco-2 cells using RNAi technology, after which cells were treated with BBM and subsequently infected with SARS-CoV-2 pseudovirus (Figure 5(A)). In Caco-2 cells transfected with control siRNA, there remained a strong preventative effect of BBM on SARS-CoV-2 acquisition (Figure 5(B)). Markedly, this antiviral effect was abrogated upon the knockdown of BNIP3 (Figure 5(B,C)), which indicates that BBM renders intestinal epithelial cells resistant to SARS-CoV-2 via a BNIP3-mediated autophagy blockade.

Finally, we demonstrated that by blocking autophagy flux, we can suppress the replication of SARS-CoV-2 Omicron BA.2 (Figure 5(D,F)) and BA.5 (Figure 5(E,G)) subvariants in Caco-2 cells in a dose–response curve, determined by high-content microscopy analyses. Pre-treatment with BBM for 3 h prior to infection robustly reduced both SARS-CoV-2 nucleocapsid and dsRNA signals, without introducing cytotoxicity and with nanomolar activity (Figure 5(D-G)). Markedly, EC_50_ values (half-maximal effective concentration; drug concentration required to reduce infection by 50%) against SARS-CoV-2 Omicron BA.2 were 4 nM or 3 nM (Figure 5(F)), whereas for Omicron BA.5, they were 0.5 nM or 1.3 nM, as measured by nucleocapsid or dsRNA staining, respectively (Figure 5(G)). Inhibition of the TMPRSS2-dependent cell-surface entry route by pre-treatment with camostat mesylate also reduced the replication of SARS-CoV-2 Omicron BA.5. Notably, in contrast to treatment the autophagy blocker BBM, TMPRSS2 inhibition exhibited only micromolar antiviral activity, with EC_50_ values of 0.2 µM for both nucleocapsid and dsRNA (Figure 5(H)), which is consistent with previously published values for SARS-CoV-2 Omicron [54]. Hence, these data corroborate the superior capacity of autophagy-based host-directed therapeutics, as exemplified by autophagy blocker BBM, to restrict the dissemination of the current dominating Omicron subvariant BA.5 at nanomolar potency.

Together, these data indicate that host autophagy is hijacked by SARS-CoV-2 to establish intestinal infection and underscore the therapeutic potential of targeting the endosomal/autophagy entry route, in particular via BNIP3-based strategies directed at autophagosome-lysosome fusion, to suppress intestinal SARS-CoV-2 entry and replication as well as limit SARS-CoV-2-mediated intestinal barrier damage. Thus, host-directed antiviral therapeutics focusing on autophagy mechanisms hold potential to limit extrapulmonary SARS-CoV-2 dissemination and gastrointestinal manifestations associated with COVID-19 disease as well as post-COVID conditions [10]. Further longitudinal studies are necessary to establish the role of targeting autophagy mechanisms in reducing and preventing post-COVID conditions.

## Discussion

Autophagy not only plays a crucial role in maintaining intestinal homeostasis, but is also implicated in viral infectious diseases. Depending on the type of virus and the viral components present at particular steps of the replication cycle, host autophagy can have either proviral or antiviral functions. In the case of coronavirus replication cycles, the intimate interplay between autophagy and the endolysosomal system is particularly interesting [6,29]. Although high autophagy flux can exert antiviral function by promoting autophagic degradation of virions, early steps of the autophagy pathway are utilized during endosomal SARS-CoV-2 entry, which is preferentially used by Omicron subvariants BA.1 and BA.2, and to a lesser extent BA.5 [6,29,30]. We have previously demonstrated that the utilization of autophagy-modulating drugs is an effective strategy for limiting viral entry and replication in primary human cells, underlining host autophagy as a relevant target for host-directed antiviral therapies [17]. COVID-19 disease pathogenesis and post-COVID syndrome is associated with several extrapulmonary organ-specific manifestations including gastrointestinal symptoms [10,13,55]. Herein, we used intestinal epithelial cell models to investigate the therapeutic potential of autophagy-targeting therapies against extrapulmonary SARS-CoV-2 dissemination. We envision that autophagy-targeting therapies could be implemented as repurposed drugs or in combination with other antivirals or host-directed therapeutics in a clinical setting to intervene in COVID-19.

Notably, we show that the knockdown of SNAP29, which is necessary for the fusion of lysosomes with other intracellular vesicles [47], reduced SARS-CoV-2 infection, indicating that autophagosome-lysosome fusion underlies the intracellular route for SARS-CoV-2 entry into intestinal epithelial cells, in line with studies in the airway and other cell line models (Figure 2(C)) [6]. In concordance with previous reports, inhibition of TMPRSS2 surface serine proteases with camostat mesylate also resulted in reduced SARS-CoV-2 infection of epithelial cells via the surface receptor entry route (Figure 2(B)). Taken together, this illustrates that both cell-surface and endosomal/autophagy routes are utilized by SARS-Cov-2 for entry into intestinal epithelial cells.

Having confirmed a role for autophagy in the infection of intestinal epithelial cells, we demonstrated that treatment with autophagy flux blockers BBM or DAS inhibited SARS-CoV-2 pseudovirus entry, corroborating the importance of an acidic intravesicular environment for SARS-CoV-2 infection (Figure 3(I,J)). Strikingly, BBM exhibited intestinal antiviral activity at as low as nanomolar concentrations against SARS-CoV-2 Omicron subvariants BA.2 and BA.5 (Figure 5(D-G)) in an autophagy-mediated BNIP3 mechanism (Figures 5(B,C)). These data underscore the potential of autophagy-modulating drugs to intervene in SARS-CoV-2 variant infections, and lay the ground for further studies to investigate the effect of these drugs on primary SARS-CoV-2 target organ(oids), as well as other secondary target organs.

Furthermore, our data also demonstrate that exposure to SARS-CoV-2 disrupts the barrier integrity of primary human intestinal monolayers (Figure 1(H)), which is consistent with previous *in vivo* data identifying increased apoptotic epithelial cells in the duodena alongside intestinal damage, resulting in microbial translocation in COVID-19 patients [13]. Disruption of the intestinal epithelial barrier might not only amplify the dissemination of SARS-CoV-2 from the gastrointestinal tract to extraintestinal sites, but also contribute to other abnormalities observed in patients with post-acute COVID-19 syndrome, such as intestinal dysbiosis and maladaptive neuro-immune interactions in the gastrointestinal compartment [10]. Although it is well established that extrapulmonary dissemination upon COVID-19 infection can lead to widespread endothelial cell damage, dysregulated immune responses, and excessive release of pro-inflammatory cytokines [9,10], thus far investigation into the mechanisms and potential treatments of gastrointestinal SARS-CoV-2 infection and virus-mediated tissue damage have largely focused on the ACE2/TPMRSS2-mediated entry route. It has been suggested that the interaction of SARS-COV-2 with ACE2 in IECs alters tight junction functioning contributing to the loss of barrier integrity [21]. In addition, binding of SARS-CoV-2 to ACE2 in enterocytes possibly results in reduced activation of canonical autophagy regulator mTOR, thereby enhancing intestinal autophagy and promoting the development of extrapulmonary COVID-19 clinical manifestations such as diarrhoea [55]. Autophagy blocking therapeutics could therefore be utilized to re-equilibrate intestinal autophagy upon SARS-CoV-2 infection to reduce lingering gastrointestinal symptoms. Concordantly, our study shows that autophagy blockers readily restored intestinal monolayer barrier function (Figure 4(A,B)), highlighting the potential of targeting the endosomal entry pathway of SARS-CoV-2 in order to intervene in COVID-19-associated gastrointestinal pathogenesis.

In summary, our findings underscore the relevance of autophagy-based host-directed therapeutics to intervene in emerging viral infections [16]. In particular, we highlight the efficacy of BBM in preventing SARS-CoV-2 infection. We demonstrate that BBM treatment is effective in limiting SARS-CoV-2-induced disruption of primary intestinal monolayers (Figure 4(A,B)), as well as preventing intestinal infection with either SARS-CoV-2 pseudovirus bearing the ancestral spike protein (Figure 3(I,J)) or SARS-CoV-2 Omicron subvariants BA.2 (Figure 5(D,F)) and BA.5 (Figure 5(E,G)), in a BNIP3-dependent manner (Figure 5(B,C)).

Previous reports have highlighted that BBM treatment not only blocks autophagy flux [47], but also reduces ACE2 levels at the plasma membrane [56], and exhibits anti-inflammatory properties via inhibition of NFΚB and MAPK signalling pathways [52]. In addition, a recent study has identified BBM as a blocker of SARS-CoV-2 Spike-mediated membrane fusion of the virus to the cell [57]. The reduced surface ACE2 expression levels and decreased viral fusion upon BBM treatment, might further contribute to the BBM-mediated antiviral activity observed in human intestinal epithelial cells. Our study adds to this data in support of using BBM as a broad-spectrum antiviral, demonstrating for the first time its efficacy in blocking SARS-CoV-2 acquisition in primary human cells, and highlighting the role of its BNIP3-dependent autophagy inhibition in the protection of SARS-CoV-2 dissemination. The continuous rise of new Omicron subvariants, which have enhanced resistance to neutralizing antibodies [58,59], underscores the relevance for the use of alternative host-directed, autophagy-modulating therapeutics, to which development of antiviral resistance is unlikely [16]. As the currently highly transmittable subvariants XBB1.5 and BQ.1.1 are respectively derived from BA.2 and BA.5, we anticipate that, considering the pan-antiviral activity presented herein, BBM will also confer protection against these novel Omicron subvariants.

Looking forward, we envision the practical use of autophagy-targeting therapies in combination with other host- and virus-targeted therapeutics, such as serine protease inhibitors [18], replication inhibitors [60], or therapeutic antibodies [7], in order to concomitantly curb extrapulmonary SARS-CoV-2 dissemination and mitigate COVID-19 clinical manifestations, with limited risk of antiviral drug resistance mutations in Omicron subvariants and future emerging VOCs.

## Supplementary Material

Supplemental Material

Supplemental Material
