# Supplementary figures and images for: Berbamine suppresses intestinal SARS-CoV-2 infection via a BNIP3-dependent autophagy blockade
Source: Emerg Microbes Infect. 2023 Apr 17;12(1):2195020. doi: 10.1080/22221751.2023.2195020 (PMC10114999; doi:10.1080/22221751.2023.2195020)

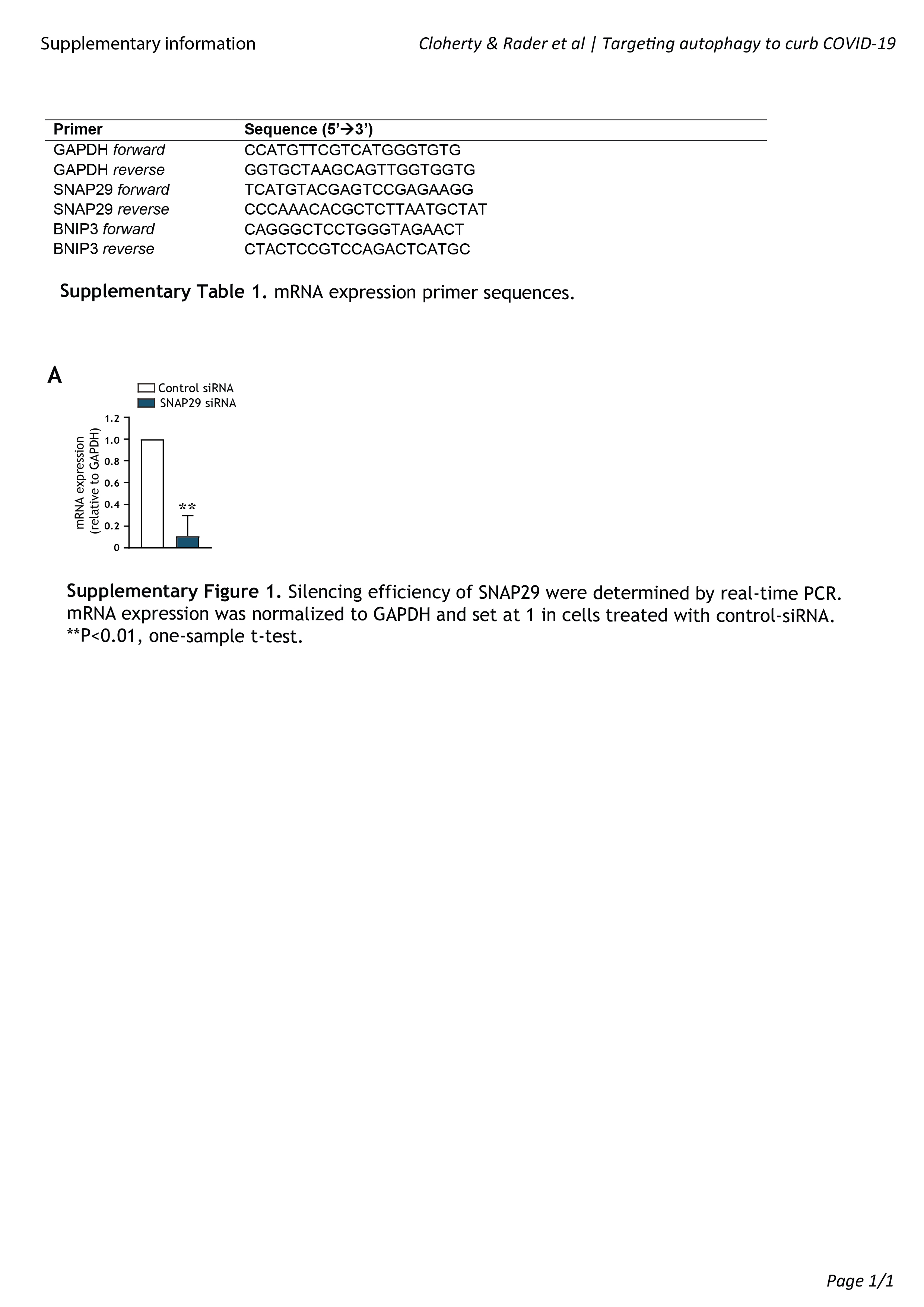

Supplement: Supplemental Material [file TEMI_A_2195020_SM4004.jpg]
