# Supplementary material for: Berbamine suppresses intestinal SARS-CoV-2 infection via a BNIP3-dependent autophagy blockade
Source: Emerg Microbes Infect. 2023 Apr 17;12(1):2195020. doi: 10.1080/22221751.2023.2195020 (PMC10114999; doi:10.1080/22221751.2023.2195020)
Supplement: Supplemental Material [file TEMI_A_2195020_SM4001.docx]

***M1. Intestinal organoid culture***

Human fetal intestinal intestines were cut open longitudinally and cut into 0.5x0.5 mm pieces and extensively washed with ice-cold PBS. Subsequently, the epithelial layer was detached by digesting the intestinal tissue pieces in PBS supplemented with 5 mM EDTA (Sigma-Aldrich), 2 mM DTT (Sigma-Aldrich) and 1% FCS (Bio-connect) for 30 minutes at 4°C. The supernatant was strained through a 70 µm cell strainer to obtain a single cell solution. Next, the single cells were washed once with PBS and then once with Advanced DMEM/F12 (Thermo Fisher Scientific) supplemented with 10 mM GlutaMAX (Thermo Fisher Scientific), 10 mM HEPES (Sigma), and 1X Penicillin/Streptomycin (Thermo Fisher Scientific), called Advanced+++ from herein. The single cells were suspended in a 1:3 Advanced+++: Matrigel (Corning) mixture and seeded in a 24-well plate (Corning) at three 10 µL droplets per well and 0.5 ml medium was added. Gut organoid cultures were maintained in Intesticult organoid growth medium (OGM; STEMCELL Technologies) components ‘Human Basal Medium’ and ‘Organoid Supplement’ in a 1:1 ratio supplemented with penicillin/streptomycin (10 U/ml and 10 μg/ml, respectively; Thermo Fisher Scientific). Medium was additionally supplemented with 10 μM Y-27632 (RHO/ROCK pathway inhibitor; STEMCELL Technologies) for the first 3 days after seeding. Medium was refreshed every 2-3 days and organoids were passaged once per week by mechanical disruption. Gut organoid cultures were kept at 37°C and 5% CO_2_.

***M2. FITC-dextran paracellular permeability assay***

Intestinal epithelial monolayer permeability was assessed using FITC-conjugated dextran (4kDa FD4, Sigma Aldrich) translocation from the apical to the basolateral compartment. Monolayer cultures were washed with Hanks’ Balanced Salt Solution without phenol-red (HBSS, Lonza) and incubated apically with 1ml/ml FITC-dextran solution for 4 h. The amount of FITC-dextran in both apical and basolateral chambers was determined using a BioTek Synergy HT plate reader. FD4 concentrations were determined using standard curves. Permeability is expressed as FD4 permeation rate: FD4 basolateral^t=4^(μg)/FD4 apical^t=0^(μg).

***M3. ACE2 expression***

Sections of infant human intestinal tissue samples were fixed with formalin and embedded in paraffin blocks prior to being cut into 5 paraffin-embedded 5 µm sections and placed in slides. Sections were then dewaxed and rehydrated, after which they were stained in an automatic immunostainer (Benchmark Ultra; Ventana Medical Systems) for ACE2 (primary: R&D Systems AF933; secondary: donkey anti-goat IgG (H+L)-Alexa Fluor 488, Invitrogen A-11055). Cell nuclei were counterstained with DAPI (Invitrogen). Tissue slides were visualized using a LEICA (DM IRB) inverted microscope and ImageJ was used to analyze the signals.

Alternatively, single cells were obtained from gut organoid cultures as described above, washed thoroughly with PBS, and stained with goat anti-ACE2 (R&D Systems AF933) followed by donkey anti-goat IgG (H+L)-Alexa Fluor 488 (Invitrogen A-11055). ACE2 expression was quantified using flow cytometry (FACSCanto II, BD Biosciences) and analysed using FlowJo (version 10.8, Treestar).

***M4. Cell line culture conditions***

The Caco-2 cell line was maintained in EMEM (ATCC #30-2003) either with 8% FCS (Bio-connect), 2mM L-glutamine (Lonza), 1x non-essential amino acids (SanBio SCC0823), and penicillin/streptomycin (10 U/ml and 10 μg/ml, respectively; Invitrogen). Caco-2 cells used in Omicron BA.2 and BA.5 infection assays were maintained in EMEM (ATCC#30-2003) with only 20% FBA (Thermo Scientific) and no additional antibiotics or supplements.

The U87.CD4.CCR5 and U87.LC3-mCherry-GFP cell lines were maintained in Iscoves Modified Dulbecco's Medium (IMDM, Thermo Fischer Scientific, USA) supplemented with 10% FCS and penicillin/streptomycin (10 U/ml and 10 μg/ml, respectively; Invitrogen).

***M5. Cell viability***

Cell viability was measured using the ATP-based CellTiter-Glo 3D reagent (Promega) according to the manufacturer’s instructions. Briefly, the CellTiter-Glo 3D reagent and white 96-well plate were equilibrated to RT. Medium was removed from the cells and 100 ul of 1:1 ratio of CellTiter-Glo 3D reagent and dPBS (Lonza) was added to the cells and mixed vigorously. After incubating for 15 minutes at 37°C to induce cell lysis, the suspension was transferred to the white 96-well plate and luminescence was measured by the SynergyHTX (BioTek) plate reader.

***M6. SARS-CoV-2 pseudovirus production***

Single-round infection pseudotyped SARS-CoV-2 (NL4.3-ΔENV-luc-GB-pseudo-SARS-CoV-2-SpikeΔ21) were generated as described previously [50,51]. Briefly, plasmid amplification was achieved by transformation into DH5α or Stbl3 *E. coli* bacteria (Invitrogen), respectively, and subsequent co-transfection of plasmids was performed in human embryonic kidney (HEK)-293T cells using a GeneJuice (Novagen, USA) transfection kit, according to manufacturer instructions. Virus was collected 2 days after transfection and filtered over a 0.2µM nitrocellulose membrane (Sartorius Stedim, Gottingen, Germany). SARS-CoV-2 pseudovirus titre was determined by luciferase reporter activity after 3 days of infection in Human embryonic kidney 293 (HEK-293T) cells expressing human ACE2 (HEK-293T-hACE2) obtained through BEI Resources, NIAID, NIH: Human Embryonic Kidney Cells (HEK-293T) Expressing Human Angiotensin-Converting Enzyme 2, HEK-293T-hACE2 Cell Line, NR-52511. Stock titers (tissue culture infection dose 50, TCID50) were determined by titration on Vero cells.

***M7. SARS-CoV-2 Omicron BA.2 and BA.5 production***

All infections with replicative SARS-CoV-2 viruses were carried out in the UBC FINDER or SFU BIO3 Biosafety Level 3 (BSL3) facilities in accordance with the Public Health Agency of Canada and UBC FINDER or SFU BIO3 regulations (UBC BSL3 Permit # B20-0105 and SFU Permit #361-2021). SARS-CoV-2 Omicron BA.2 was kindly provided by Dr. Mel Krajden (BC Centre for Disease Control, BC, Canada). SARS-CoV-2 Omicron BA.5 was isolated by Dr. Masahiro Niikura (SFU) from a clinical specimen in VeroE6/TMPRSS2 cells and confirmed as a BA.5 variant by complete genome sequencing (sequence available in GISAID). Omicron subvariants BA.2 and BA.5 were amplified in VeroE6/TMPRSS2 cells and used in the experiments at passage 2.
